# Top-down modulation of attention by emotion

**DOI:** 10.3389/fnhum.2013.00102

**Published:** 2013-04-01

**Authors:** Aprajita Mohanty, Tamara J. Sussman

**Affiliations:** Department of Psychology, Stony Brook UniversityStony Brook, NY, USA

Due to their evolutionary salience, threat-related stimuli, such as snakes, spiders, and angry faces constitute a special class of stimuli believed to capture attention in an involuntary, bottom-up manner. Most research in affective neuroscience has focused on unraveling neural pathways that support this “automatic” capture of attention by emotional stimuli (Vuilleumier and Driver, 2007). However, it is well known that in addition to stimulus-driven bottom-up factors (Itti and Koch, 2001), attention is guided by goal-driven, top-down factors (Hopfinger et al., 2000) such as anticipated locations and features of upcoming targets (Moran and Desimone, 1985; Treue and Martinez Trujillo, 1999). In real life, we often utilize emotional information endogenously to guide our attention, for example, when looking for cars while crossing a street or for a restaurant when hungry. These anticipatory search behaviors, aimed at detecting sources of potential threat or reward are deployed in a wide range of habitats from the savannah to social gatherings. Below, we review behavioral and neural data that highlight the importance of emotional factors in top-down voluntary guidance of attention. Based on these findings, we espouse a shift in emphasis from examining emotional factors as primarily impacting attention in a bottom-up manner to examining them in an endogenous, voluntary role wherein emotional information is strategically utilized to guide perception and attention. Cognitive behavioral formulations of anxiety have proposed an important role for threat-related schemata in the development and maintenance of anxiety (Beck, 1976; Mogg et al., 1989). In light of this, research examining the role of expectation and anticipatory attention toward threat will contribute not only to a more comprehensive understanding of normal emotion-attention interactions but also to our understanding of the development and maintenance of anxiety.

## Bottom-up capture of attention by emotional stimuli

To deal with the overwhelming excess of incoming information, the visual system utilizes mechanisms that bias the competition between stimuli toward preferential representation of the most relevant subset of stimuli (Desimone and Duncan, 1995). This biasing process consists of two attentional mechanisms: a bottom-up sensory driven mechanism that biases selection of stimuli based on their physical saliency, and a top-down mechanism, which directs attention endogenously under volitional control. In contrast to the top-down mechanisms, bottom-up attention mechanisms are thought to operate by involuntarily or exogenously shifting attention to salient visual stimuli. For example, stimuli that create a local discontinuity in the visual environment, such as abrupt occurrence of a new object (Jonides and Yantis, 1988), sudden motion (Abrams and Christ, 2003; Franconeri and Simons, 2003), looming, and luminance contrast changes (Enns et al., 2001) are given more attentional priority. Emotional stimuli are another class of stimuli believed capture attention involuntarily (Ohman et al., 2000; Ohman and Mineka, 2001). For example, in visual search arrays, angry faces are detected faster and more efficiently than neutral and happy faces (Eastwood et al., 2001; Tipples et al., 2002) and attentional probes appearing in the same location as threatening faces are detected faster than probes appearing in the opposite location (Mogg and Bradley, 1999; Armony and Dolan, 2002; Pourtois et al., 2004). It remains unclear if the bottom-up capture of attention by an emotional stimulus such a threatening face is driven by specific physical features of the stimulus such as a downward pointing “V,” which is similar to the geometric configuration of the face in angry expressions (Larson et al., 2008) or by complex interactions between facial feature configurations and elicited emotion (Lundqvist and Ohman, 2005).

The literature on the impact of emotion on attention has been biased toward examining emotion in a bottom-up role, for example, when attention is captured by an emotional stimulus that “pops out” in a crowd of non-emotional stimuli (Fox et al., 2000; Ohman et al., 2001) or is presented peripherally in a covert attention task (Mogg and Bradley, 1999; Armony and Dolan, 2002), or creates emotion-induced blindness to a preceding or succeeding target in a stream of images (Most et al., 2005), or is the irrelevant to the task (Williams et al., 1996; Algom et al., 2004). This involuntary capture of attention by emotion-related information is hypothesized to involve amygdala and orbitofrontal cortex mediated modulation of visual processing (Anderson and Phelps, 2001; Bar et al., 2006; Vuilleumier and Driver, 2007; Lim et al., 2009) and is considered independent of attention-related frontoparietal modulation of visual processing (Vuilleumier and Driver, 2007), although there is evidence that activity in this network is modulated by attentional demands (Lim et al., 2009).

## Top-down modulation of bottom-up attentional capture by emotional stimuli

Considerable research has shown that bottom-up capture of attention by emotional stimuli and related neural mechanisms, including amygdala and its influence on the visual cortex, is susceptible to top-down factors like task-context and attentional control (Pessoa, 2008; Pessoa and Adolphs, 2010). In addition to these cognitive top-down factors, emotional/motivational top-down factors (e.g., searching for threat or anticipating reward) can modulate bottom-up capture of attention. For example, happy and threatening facial expressions capture attention when they are the target of search (Williams et al., 2005; Hahn and Gronlund, 2007) but not when they are in opposition to task goals, indicating that in addition to stimulus characteristics, emotion-related top-down goals guide the efficiency of facial expression search. Reward contingencies associated with different targets influence priming of pop-out, measured as improved search performance for pop-out targets (e.g., red among green) that are repeated vs. non-repeated on successive trials, indicating a motivational top-down influence of goals on a phenomenon considered sensitive only to bottom-up manipulations (Kristjansson et al., 2010). Reward, promise of reward and punishment are associated with greater perceptual sensitivity on an exogenous spatial attention task (Engelmann and Pessoa, 2007) and greater distractor inhibition (Della Libera and Chelazzic, 2006). In a spatial attention task, words associated with temporary goals hold attention longer than semantically related words, suggesting that these goals influence the allocation of attention (Vogt et al., 2010). Following disgust induction, participants orient toward pictures representing disgust and cleanliness indicating that, in addition to being stimulus-driven, deployment of attention is guided by the goal to alleviate the aversive state (Vogt et al., 2011).

This competition between bottom-up and top-down factors is explicated by the arousal-biased competition (ABC) model of attention which proposes that emotional arousal related to a top-down goal or state can increase attention toward high priority information, while diminishing attention toward low priority information, regardless of whether the information has priority because of its bottom-up attention grabbing nature or top-down goals, expectations, or contexts (Mather and Sutherland, 2011).

## Top-down guidance of attention by emotional cues

Increasingly, research is showing that emotional information can be employed endogenously to guide attention. Studies are beginning to elucidate the psychological and neural mechanisms involved in anticipatory biasing of attention by threat or reward-related cues. These studies utilize functional neuroimaging and covert attention paradigms wherein attention is engaged voluntarily (“endogenously”) via a central cue directing attention toward expected peripheral locations of salient attentional targets (Small et al., 2005; Mohanty et al., 2008, 2009). It is well-established that that the network of brain regions involved in sensory-motor aspects of top-down spatial biasing of attention include posterior parietal cortex (PPC), including intraparietal sulcus (IPS) and extending to inferior and superior parietal lobule (IPL/SPL), lateral frontal cortex, including the frontal eye fields (FEF), and cingulate gyrus, including its anterior (AC) and posterior (PC) segments (Mesulam, 1981, 1999; Corbetta and Shulman, 2002). Recent research on the top-down guidance of attention by emotional cues has focused on understanding how limbic and dopaminergic regions that encode motivational salience of attentional cues interact with the frontoparietal spatial attention network that guides attention toward salient attentional targets.

**Figure 1:**
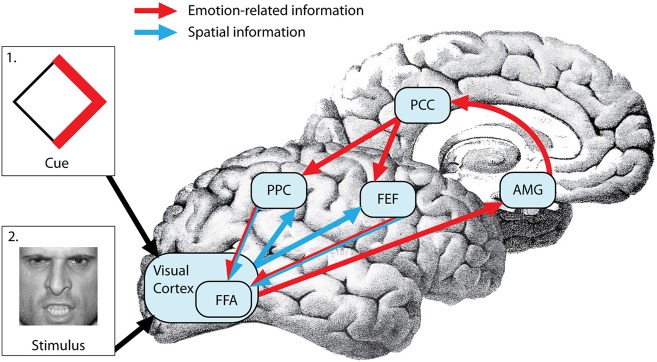
**Neural mechanisms of top-down emotional modulation of attention**. Prior information regarding upcoming attentional targets (via cue depicted in box 1) is processed in the visual cortex (VC). Emotion-related information from the VC is evaluated in the amygdala (AMG) and projected to the posterior parietal cortex (PPC) and frontal eye fields (FEF) through the post cingulate cortex (PCC). On the other hand, spatial information encoded in the cue is projected from the VC to the FEF and PPC. The FEF and PPC form an integrated search template (a “top-down salience map”) that combines the spatial coordinates of the an event with its emotional salience and bias the visual cortex (more specifically fusiform face area or FFA in this case because the attentional target is a face) in preparation for the search process resulting in faster detection of the target (depicted in box 2).

While it is clear that limbic and frontoparietal regions are involved in motivational guidance of attention, how exactly is prior motivational information integrated with sensory-motor components of spatial attention? One possibility is that emotional and spatial information is integrated in the amygdala, as has been shown in a recent primate study (Peck et al., 2013). An alternative possibility is that the spatial and emotion-related information is integrated by combining anatomically segregated frontoparietal and limbic inputs in the visual cortex. A third possibility is that prior access to spatial and emotional information regarding the attentional target allows the integration of these two sources of information in frontoparietal regions that provide the top-down biasing of visual cortical areas (Figure 1). The spatial attention network forms an integrated search template (a “top-down salience map”) that combines the spatial coordinates of an event with its task relevance and biases visual neurons in preparation for the search process in both humans and monkeys (Thompson et al., 2005; Gottlieb, 2007; Egner, 2008). IPL and IPS (area LIP) neurons are sensitive to the motivational value of stimuli in monkeys (Mountcastle et al., 1975; Bushnell et al., 1981; Sugrue et al., 2004) and limbic regions such as amygdala are important in assessing the motivational salience of stimuli in humans (Pessoa et al., 2002; Vuilleumier and Driver, 2007), but whether and how these regions communicate is unclear. The rostro-caudal parts of the cingulate gyrus send monosynaptic projections to frontoparietal regions and PC neurons signal reward outcomes associated with shifts of gaze (McCoy et al., 2003) and subjective preferences that guide visual orienting (McCoy and Platt, 2005) in monkeys, raising the possibility that the cingulate gyrus is the conduit for information on motivational salience used by the spatial attention network (Mesulam et al., 1977; Shackman et al., 2011).

Neural hypotheses regarding the integration of emotional and spatial information in frontoparietal brain regions (Figure 1) were tested in a study in which centrally-located cues predicted locations of peripherally presented food or tool-related attentional target images (Mohanty et al., 2008). The motivational value of the food targets was experimentally manipulated via hunger and satiety. Hemodynamic responses were measured to the central cues preceding the food targets, as opposed to the target stimuli that are typically imaged in bottom-up attention studies. Results showed increased amygdala, PC, locus coeruleus (LC), and substantia nigra (SN) activity for food-related cues when hungry but not when satiated. Since the spatial resolution of the fMRI does not allow for precise localization of small structures such as the LC and SN, caution must be used when identifying these regions. However, the activation locations found were compatible with locations reported in previous studies (O'Doherty et al., 2002; Wittmann et al., 2005). Furthermore, activation in components of the spatial attention network such as PPC, banks of the IPS, and PC was more positively correlated with the speed of attentional shifts to food targets when hungry than full. These findings indicate that anticipatory allocation of attention via spatial attention regions is sensitive not only to motivational state but also to the motivational value of the upcoming targets. Furthermore, in this study PC neurons were sensitive to the motivational valence of an upcoming stimulus, positively correlated with the speed of attentional shifts to food targets when hungry than full, and showed stronger functional coupling with IPS during spatial biasing of attention toward motivationally relevant stimuli providing support for the possibility that the PC serves as a neural interface between limbic system that encodes motivational value of upcoming targets and the frontoparietal regions that direct attention to these targets.

In another study, *endogenous* guidance of attention was manipulated by predictive cues that offered probabilistic information related to the location and emotional salience of an upcoming stimulus (Mohanty et al., 2009). This study utilized a visual cued search task in which centrally located cues provided spatial information (valid cues indicated the location of upcoming targets while uninformative cues provided no information) and emotional information (valid cues indicated the valence of upcoming targets and uninformative cues provided no information) regarding upcoming peripherally presented targets. While spatially valid cues enhanced the detection of targets, cues validly predicting threatening face targets (endogenously driven attention) resulted in faster reaction times than uninformative cues followed by threatening faces (bottom-up capture of attention), indicating that the emotional cue-related acceleration of spatial attention can be endogenously mediated and is not solely dependent on bottom-up target features. Functional imaging showed, even before the appearance of the target, spatially informative cues activated the spatial attention network including IPS and FEF, as well as fusiform gyrus (FG), whereas cues predicting angry faces also activated limbic areas, including the amygdala. Anatomically overlapping, additive effects of spatial and emotional cueing were identified in IPS, FEF, and FG. The FG also displayed augmented connectivity with the amygdala following angry face cues. These data suggest that anticipatory search for a threatening stimulus elicits amygdala input to the spatial attention network and inferotemporal visual areas, facilitating the rapid detection of upcoming motivationally significant events.

From these studies it is clear that attention can be driven endogenously by both appetitive and aversive factors. Although brain regions involved in the evaluation of motivational value of stimuli (appetitive or aversive) may be different; for example, aversive information may be evaluated in regions such as amygdala (Dolan and Vuilleumier, 2003) while appetitive information is processed in areas including the dopaminergic mid-brain and striatum (O'Doherty et al., 2002), motivational and spatial information regarding attentional targets is integrated in the frontoparietal attention network regardless of stimuli valence. Separate from the effects of attention, expectations regarding upcoming targets can enhance their perception (Summerfield and Egner, 2009). According to the “predictive coding” theory, rather than passively absorbing sensory input, the brain actively predicts what is upcoming, generating a pre-stimulus template against which observed sensory information is matched (Summerfield et al., 2006; Zelano et al., 2011). Knowledge and past experience set expectations for the likely sensory input, facilitating the speed and accuracy of subsequent perceptual judgments. Hence, the expectation of, rather than actual encounter with emotional stimuli may be a key factor in accounting for enhanced perception of these stimuli. Put another way, predictive representations of emotional stimuli might confer a distinct processing advantage compared to neutral stimuli.

In summary, it is clear that the role of emotional factors in anticipatory allocation of spatial attention has been relatively neglected. To understand how emotional factors guide spatial attention, it is necessary to consider not only how they influence involuntary shifts in attention, but also how they voluntarily shift attention toward visual targets. Furthermore, it is necessary not only to consider emotional and spatial attention effects on spatial orienting, but to assess how these effects are integrated, as well as how emotional features (Lundqvist and Ohman, 2005) may be utilized to guide attention. The examination of voluntary recruitment of attention for threat-related information may yield important clues into both the development and maintenance of anxiety. For example, this research would help clarify how top-down aspects of anxiety, such as worry, rumination, threat-based schemas, and poor attentional control contribute to the development of attentional biases to threat and ultimately contribute to development and maintenance of anxiety.
